# TLR5: A prognostic and monitoring indicator for triple-negative breast cancer

**DOI:** 10.1038/s41419-019-2187-8

**Published:** 2019-12-18

**Authors:** Dai Shi, Shanshan Zhao, Wen Jiang, Chao Zhang, Ting Liang, Guihua Hou

**Affiliations:** Key Laboratory for Experimental Teratology of the Ministry of Education and Biomedical Isotope Research Center, School of Basic Medical Sciences, Shandong University, Jinan, Shandong China

**Keywords:** Breast cancer, Toll-like receptors, Experimental models of disease

## Abstract

A novel, highly selective biomarker is urgently needed to predict and monitor triple-negative breast cancer (TNBC) because targeting molecules are not currently available. Although associated with various malignant tumors, the role of toll-like receptor 5 (TLR5) in TNBC remains uncertain. We aimed to define the effects of TLR5 in TNBC to determine whether it could serve as a prognostic and monitoring indicator for TNBC. We established TNBC cell line 4T1 with low TLR5 expression (GFP tag; TLR5^−^ 4T1) and with normal TLR5 expression (GFP tag; TLR5^+^ 4T1) using lentivirus-shRNA-TLR5 knockdown transfection and negative lentivirus transfection, respectively. Detected by western blot and qPCR, we found knockdown of TLR5 resulted in decreased expression of TLR5 and E-cadherin and increased expression of N-cadherin, vimentin, fibronectin, TRAF6, SOX2, and Twist1, which were related to EMT (epithelial–mesenchymal transition). In addition, downregulation of TLR5 increased the invasion and migration of 4T1 cells in vitro, which were investigated by CCK-8 and wound healing, as well as transwell assay and colony formation. Furthermore, the metastatic ability of TLR5^−^ 4T1 cells to the lungs was also increased compared to TLR5^+^ 4T1 cells in vivo. To verify the effect of TLR5 as a monitor indicator, mice bearing TLR5^+^ and TLR5^−^ 4T1 tumors injected with ^125^I-anti-TLR5 mAb or isotype ^125^I-IgG were assessed by whole body phosphor-autoradiography and fluorescence imaging in vivo. Phosphor-autoradiography of model mice revealed early tumors at 6 days after inoculation with TLR5^+^ 4T1, but not TLR5^−^ 4T1 cells. Intratumoral accumulation of radioactivity positively correlated with TLR5 expression, and fluorescence imaging in vivo revealed both TLR5^+^ and TLR5^−^ 4T1 tumors. Our results suggested that downregulation of TLR5 in TNBC increased tumor invasiveness and EMT expression via TRAF6 and SOX2 pathway and TLR5 could serve as a prognostic and monitoring indicator for TLR5-positive tumors.

## Introduction

Membrane-bound toll-like receptors (TLRs) play key roles in innate and adaptive immunity. They are primarily expressed in some types of immunocytes and recognize conserved pathogen-associated molecular patterns (PAMPs)^1–4^. Various members of the TLR family play diversified roles in cancer progression and development^5,6^. For example, positive TLR3 expression indicates a favorable prognosis for patients with neuroblastoma^7^, and TLR4 overexpression is reduced in squamous cell carcinoma of the skin^8^. Unlike other TLRs, TLR5 is not expressed in murine macrophages and conventional dendritic cells^9^, but it is expressed at high levels in some malignancies, such as non-small cell lung cancer^10^ and NK cells within breast cancer^11^. Cancer cell proliferation and tumor growth are inhibited by TLR5 signaling^12–14^, but the underlying mechanism has not been fully elucidated.

Breast cancer is the most common cancer among women, and it is associated with high mortality rates^15–17^. It can be classified into TNBC (triple-negative breast cancer) and non-TNBC subtypes. The TNBC type accounts for 12–24% of all breast cancers and is more aggressive and prone to metastasis than the non-TNBC type. Current clinical management of breast cancer mainly depends on estrogen receptor (ER), progesterone receptor (PR), and human epidermal growth factor receptor 2 (HER2) genes as molecular markers and therapeutic and prognostic indicators. However, targeted diagnoses and therapy are not currently feasible for TNBC because it does not express ER, PR, and HER2^18^. Radical surgery is still the first choice of treatment for breast cancer, even though metastasis usually appears within a few years^19^. Hence, the identification of new monitoring molecular markers and clarifying the underlying mechanisms are critical for improved prognosis for patients with TNBC.

Traditional computed tomography (CT) and magnetic resonance imaging (MRI) in tumor diagnosis and staging mainly reveal anatomic changes, and these are usually found at the later stages^20^. Noninvasive nuclear molecular imaging is a whole-body scanning modality that is based on metabolism and abnormal function. It is a combination of anatomical and functional imaging and is more suitable for the detection and quantitation of target molecules in tumor tissues. Presently, F-18-deoxyglucose (^18^F-FDG) positron emission tomography/computed tomography (PET/CT) is clinically applied to evaluate tumor metabolism^21^. However, ^18^F-FDG is not specific, which sometimes results in diagnostic inaccuracy. Hence, identifying more effective molecular targets for tumor prognosis and therapy is critical. Many recent studies have shown that some innate molecules participate in tumor development and prognosis, such as colon cancer-associated transcript2 (CCAT2), metastasis and prognosis^22^ and three miRNAs (potential prognostic biomarkers in patients with bladder cancer)^23^. Molecular imaging has rapidly developed and shown great potential in early tumor diagnosis and treatment based on physical and metabolic changes^24^. Radioisotope-labeled target molecules for breast cancer such as ^18^F-labeled aptamers of Her2/ErbB2 (human epidermal growth factor receptor 2)^25^, ^125^I/^131^I-labeled anti ICAM-1(intercellular cell adhesion molecule-1) antibody^26^ and ^89^Zr-Transferrin^27^, have been developed. However, these target molecules are limited by low selection and tumor expression. We developed a ^131^I-labeled anti-TLR5 antibody that can indicate allorejection^28^. Here, we aimed to determine whether this radiolabeled antibody could serve as a noninvasive monitoring target for early TNBC using the TNBC cell line, 4T1.

## Materials and methods

### Mice and reagents

All animal studies proceeded in accordance with protocols approved by the Animal Care and Use Committee of Shandong University. Six-week-old male athymic nude mice (20–22 g) were purchased from Charles River Laboratories (Wilmington, MA, USA). The Lentivirus-shRNA (5′–3′ GCTTCAACTATATCAGTATGG) for TLR5 knockdown and negative-control virus was purchased from GenePharma (Shanghai, China). Anti-vimentin (#5741), anti-N-cadherin (#13116), anti-E-cadherin (#3195), anti-SOX2 (#14962) and anti-GAPDH (#5174) antibodies were purchased from Cell Signaling Technologies (CST; Boston, MA, USA). Anti-TLR5 (#ab168382), anti-fibronectin (#ab45688), anti-TRAF6 (#ab33915) and anti-Twist1 (#ab49254) antibodies were purchased from Abcam (Cambridge, UK).

### Generation of TLR5 knockdown 4T1 cells

Murine TNBC cell lines 4T1^23^ were obtained from the American Type Culture Collection (ATCC). The cells were seeded in 24-well plates (1 × 10^5^/well) and incubated at 37 °C in a humidified 5% CO_2_ atmosphere overnight. The medium was then replaced with negative control virus or Lentivirus-shRNA TLR5 diluted in fresh medium at a ratio of 1:100, and the cells were incubated at 37 °C in a humidified 5% CO_2_ atmosphere for 24 h. Finally, the diluted virus was replaced with fresh medium and incubation proceeded under the same conditions for another 48 h. Thereafter, transfection efficiency was calculated. Transfected cells were filtered out using Puromycin (Beijing Solarbio Science & Technology Co., Ltd., Beijing, China).

### Western blot and quantitative PCR (qPCR)

Total protein concentrations for western blot were measured using protein assay kits (Beyotime Biotechnology, Shanghai, China). Total protein (20 μg) was loaded onto gel (Bio-Rad Laboratories, Hercules, CA, USA), along with Chameleon Duo ladder protein marker (SMOBIO, Taiwan, R.O.C). Proteins were resolved by electrophoresis at 80 V for 30 min followed by 100 V for 60 min and transferred onto nitrocellulose membranes. Nonspecific binding was blocked with 5% skim milk in blocking buffer for 2 h at room temperature (20 °C) and further incubated overnight at 4 °C with 1:1000 diluted rabbit anti-mouse TLR5/vimentin/fibronectin/N-cadherin/E-cadherin/TRAF6/SOX2/Twist1 antibodies and 1:10000 diluted rabbit anti-mouse GAPDH antibody. Membranes were washed three times with TBS-Tween 20 (TBS-T), incubated with anti-rabbit IgG antibodies (1:5000) for 2 h at room temperature, then scanned and quantified using a Tanon 4200 imaging system (Tanon Science and Technology Co., Ltd., Shanghai, China).

Total RNA was extracted for qPCR from 4T1 cells using the TRIzol reagent (Invitrogen) according to the manufacturer’s instructions. After determining the RNA concentration, the cDNA first strand was synthesized via TransScript First-Strand cDNA Synthesis SuperMix (TRANSGEN BIOTECH, Beijing, China). The mRNA was then measured using TransStart Tip Green qPCR SuperMix (TRANSGEN BIOTECH, Beijing, China) with the following forward and reverse primers, respectively:

TLR5, 5′-GCAGGATCATGGCATGTCAAC-3′ and 5′-ATCTGGGTGAGGTTACAGCCT-3′;

E-cadherin, 5′-CAGGTCTCCTCATGGCTTTGC-3′ and 5′-CTTCCGAAAAGAAGGCTGTCC-3′;

N-cadherin, 5′- AGCGCAGTCTTACCGAAGG-3′ and 5′-TCGCTGCTTTCATACTGAACTTT-3′;

vimentin, 5′- CGTCCACACGCACCTACAG3′ and 5′- GGGGGATGAGGAATAGAGGCT-3′;

TRAF6, 5′- TAAGGGATGCAGGGCACAAG-3′ and 5′- GGCACTTTACCGTCAGGGAA-3′;

SOX2, 5′- TTTGTCCGAGACCGAGAAGC-3′ and 5′- CTCCGGGAAGCGTGTACTTA-3′;

Twist1, 5′- GGACAAGCTGAGCAAGATTCA-3′ and 5′-CGGAGAAGGCGTAGCTGAG-3′.

### CCK-8 assays

Cell proliferation was evaluated using CCK-8 assays. TLR5^+^ and TLR5^−^ 4T1 cells (2 × 10^3^ well) were, respectively, seeded in 96-well plates overnight, and the absorbance was measured at 450 nm after 0, 6, 24, 36, and 48 h by using a microplate reader. Experiments were repeated three times.

### Flow cytometry

Apoptosis was evaluated by digesting TLR5^+^ and TLR5^−^ 4T1 cells with 0.25% trypsin (without EDTA and phenol red), after which, the cells were sedimented by centrifugation (speed in 800*g*, for 5 min). The cells were washed twice with cold PBS and suspended in 400 μL of 1 × annexin V binding solution at a concentration of 1 × 10^6^/mL. Annexin V-YF647A (5 μL) was gently mixed with 10 μL of PI staining solution, incubated at 4 °C for 15 min in darkness, passed through a 200-mesh filter, and immediately analyzed by flow cytometry. Experiments were repeated three times.

### Colony formation

TLR5^+^ and TLR5^−^ 4T1 cells (1 × 10^3^/well) were seeded in six-well plates and the medium was changed every 3–4 days. The plates were incubated at 37 °C in a humidified incubator for 14 days. At the end of the experiment, the cells were washed with PBS, incubated with 0.005% crystal violet for 15 min, then rinsed with PBS. Colony formation rates were calculated as follows: (number of colonies/number of seeded cells) × 100%. Experiments were repeated three times.

### Wounding healing assays

TLR5^+^ and TLR5^−^ 4T1 cells were seeded in six-well plates and incubated overnight to obtain confluent monolayers. Monolayers were scratched using a sterile pipette tip, and wounds were examined after incubation at 37 °C in a humidified 5% CO_2_ incubator for 24 h. Experiments were repeated three times.

### Transwell assays

These assays proceeded in 24-well Transwell units with an 8 μm pore polycarbonate membrane. Matrigel (100 μL; 300 μg/mL) was added to the upper compartment. After overnight starvation, suspended cells were seeded in the upper compartment in serum-free medium, and medium supplemented with 10% FBS as a chemoattractant was placed in the lower compartment. Cells in the upper compartment were removed 24 h later by gentle swabbing. Cells that had migrated to the lower surface of the membrane were stained with crystal violet and counted at 400× magnification in five high power fields. Triplicate samples were tested, and the experiment was repeated three times.

### Lung metastasis experiment

Mice were injected with 1 × 10^6^ TLR5^+^ or TLR5^−^ 4T1 cells via the tail vein, and the mouse lungs were weighed 20 days later and dissected for hematoxylin and eosin (H&E) staining and fluorescence imaging. The experiment was repeated at least three times.

### 4T1 breast cancer-bearing mice model

All animal studies proceeded in accordance with protocols approved by the Animal Care and Use Committee of Shandong University. Subcutaneous TLR5^+^ and TLR5^−^ 4T1 tumor cells were induced in 6-week-old nude male mice, respectively, by injecting their lower left and right flanks with 1 × 10^6^ TLR5^+^ 4T1 and TLR5^−^ 4T1 cells suspended in 200 μL of PBS. The tumors on mice were monitored every other day. The tumor-bearing mice were assessed by phosphor-autoradiography and fluorescence imaging, and biodistribution was also determined.

### Preparation of ^125^I-anti-TLR5 mAb and ^125^I-IgG

We prepared ^125^I-anti-TLR5 and ^125^I-IgG as described previously^28^. A 1:2 (v/v) mixture of 0.9% saline and methanol served as an unfolding agent. The stability of ^125^I-anti-TLR5 mAb and ^125^I-IgG in PBS was evaluated using paper chromatography in PBS and murine serum, then radioactivity was determined using a Gamma counter. Competitive binding was assessed in TLR5^+^ 4T1 cells (5 × 10^5^/well) seeded in 24-well plates with 0.1–1000 nM anti-TLR5 mAb and 10 nM ^125^I-anti-TLR5 mAb and incubated at 37 °C for 45 min. The supernatants were discarded, and then the cells were washed twice with iced 1 × PBS containing 0.1% BSA and harvested for radioactivity determination using a Gamma counter.

### Dynamic whole-body phosphor-autoradiography

Three days before radiotracers injection, 3% potassium iodide was added to the drinking water of the mice to block iodine uptake by the thyroid gland. Tumor-bearing mice (total *n* = 50, *n* = 25 per group, randomly divided) were each injected with 0.37 MBq of ^125^I-antiTLR5 mAb (0.38 μg) or ^125^I-IgG (0.38 μg) via the tail vein. Whole-body phosphor-autoradiography scanning proceeded on day 6, 8, 10, 12, and 14 after tumor cell inoculation (at 48 h after the ^125^I-antiTLR5 or ^125^I-IgG injections), respectively. For the blocking group (*n* = 5, randomly divided), mice were injected with unlabeled anti-TLR5 mAb (100 μg) 30 min before the ^125^I-antiTLR5 mAb (0.37 MBq, 0.38 μg) injection. Whole-body phosphor-autoradiography scanning proceeded on day 12 after tumor cell inoculation (at 48 h after the ^125^I-antiTLR5 injections). Mice anesthetized with sodium pentobarbital (0.6%) were placed supine on a storage phosphor screen plate (back to the plate) and left for 20 min in darkness. Plate was then transferred to a Cyclone Plus scanner (PerkinElmer). Manually drawn rectangular regions of interest (*n* = 5) within the target area at each time point were semi-quantified. Digital light units (DLU)/mm^2^ were obtained using OptiQuant^TM^ image analysis software 5.0 (PerkinElmer). Tumors were individually stripped and imaged.

### Fluorescence imaging

The 4T1 cells were transfected with lentivirus-TLR5 knockdown, and the lentivirus expressed green fluorescent protein (GFP) after integration into 4T1 cells. Subcutaneous TLR5^−^ and TLR5^+^ 4T1 tumors were induced as described above with TLR5^−^ 4T1 and TLR5^+^ 4T1 cells injected into the lower right and left flanks, respectively, and 4T1 cells without Lentivirus transfection were injected into the back. The mice were anesthetized on day 6, 8, 10, 12, and 14 after tumor cell inoculation, respectively, then the skin on the surface of the tumors was peeled, and the mice were placed prone on the imaging plate and photographed using IVIS Spectrum (PerkinElmer).

### Biodistribution studies

Three days before injecting the radiotracers, 3% potassium iodide was added to the drinking water of the mice to block iodine uptake by the thyroid gland. Model mice (total *n* = 30, *n* = 15 per group, randomly divided) were injected with ^125^I-anti-TLR5 mAb or ^125^I-IgG (0.37 MBq), then sacrificed, and dissected 24, 48, and 72 h later. Tumors, blood, and major tissues/organs (heart, lung, liver, kidney, spleen, small intestine, and muscle) were harvested and weighed. Samples and primed standards were measured using a Gamma counter. Tissue radioactivity was expressed as the percent injected dose per gram (%ID/g), and the target to non-target (T/NT) ratio was defined as the ratio of radioactivity that accumulated in tumors to that in the contralateral muscle. The experiment was repeated three times.

### H&E and immunohistochemical staining

Tumor-bearing mice (*n* = 5) were sacrificed 72 h after completing whole-body phosphor-autoradiography. Tumors were isolated for immunohistochemical staining with rabbit polyclonal TLR5 antibody (Beijing Biosynthesis Biotechnology Co., Ltd., Beijing, China) and DAB chromogen (Biogenics Inc., Napa, CA, USA). Immunohistochemistry proceeded using SP-9002 Histostain^TM^ Plus kits (ZSGB-BIO, Beijing, China) according to the manufacturer’s protocols. Sections on slides were visualized at 200× and 400× magnification. Corresponding positive areas of sections were analyzed (five fields per slide) using Image-Pro Plus software version 4.5.0.29 (Media Cybernetics Inc., Rockville, MD, USA). The experiment was repeated five times.

### Statistical analysis

Data were presented as the means ± standard deviation (SD) derived from at least three independent experiments. Student *t* tests were applied using Graph Pad Prism version 5 (GraphPad Software Inc., La Jolla, CA, USA). Significant differences were considered at *P < 0.05 and **P < 0.01.

## Results

### TLR5 downregulation prompted epithelial–mesenchymal transition (EMT) in 4T1 cells

**Fig. 1:**
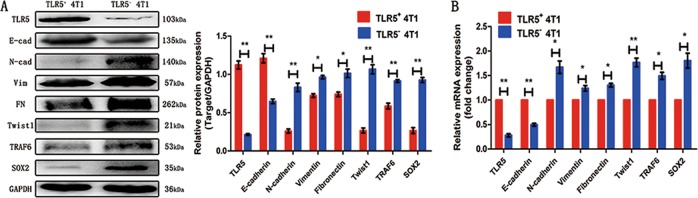
TLR5 downregulation promoted EMT in 4T1 cells. TLR5, E-cadherin, N-cadherin, vimentin, fibronectin,TRAF6, SOX2 and Twist1 protein expression of TLR5^+^ 4T1 and TLR5^−^ 4T1 cells was detected by western Blot (*n* = 3, *P < 0.05) (**a**). TLR5, E-cadherin, N-cadherin, vimentin, fibronectin, TRAF6, SOX2 and Twist1 mRNA expression of TLR5^+^ 4T1 and TLR5^−^ 4T1 cells was detected by qPCR (*n* = 3, *P < 0.05) (**b**). Representative results of three independent experiments were reported. The data were presented as the means ± SD from three independent experiments, analyzed by Student’s *t* test. *P < 0.05, **P < 0.01.

We quantified the protein and mRNA expression of TLR5, E-cadherin, N-cadherin, vimentin, fibronectin, TRAF6, SOX2 and the transcription factor, Twist1, by western blot and qPCR to verify the effects of TLR5 on EMT (Fig. 1a and b, respectively). The protein expression of E-cadherin significantly decreased after TLR5 downregulation, whereas those of vimentin, fibronectin, N-cadherin, TRAF6, SOX2 and Twist1 increased. The trend in mRNA levels was similar. These results suggested that TLR5 downregulation induced EMT increasing via TRAF6 and SOX2 pathway in TNBC.

### TLR5 downregulation prompted 4T1 tumor cell proliferation, colony formation, and migration in vitro

**Fig. 2:**
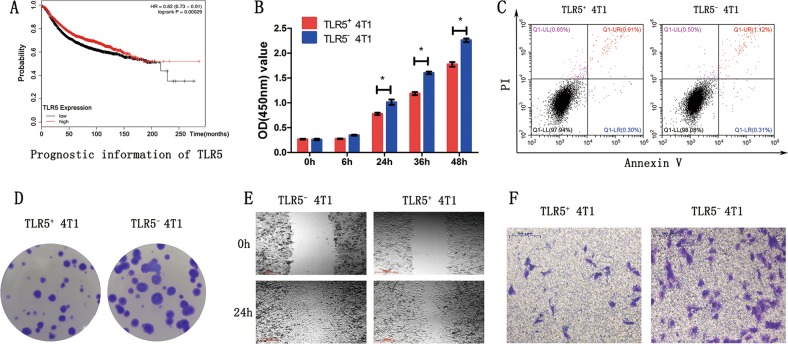
TLR5 downregulation enhanced 4T1 tumor cell proliferation, migration and invasiveness in vitro. Kaplan–Meier plotter online tools were used to identify that the patients suffered from breast cancer with low TLR5 expression had a significantly worse survival rate (**a**). CCK-8 assay showed that TLR5^−^ 4T1 cells have higher proliferation ability than TLR5^+^ 4T1 cells (**b**).No difference in apoptosis change in TLR5 downregulated 4T1 cells was detected compared with negative control group (**c**). The colony formation ability of TLR5^−^ 4T1 cells was significantly increased than TLR5^+^ 4T1 cells (**d**).The migration area of TLR5^−^ 4T1 group was larger than TLR5^+^ 4T1 group (**e**). The number of migrated cells was remarkably reduced in TLR5^+^ 4T1 group than TLR5^−^ 4T1 group (**f**). The data were presented as the means ± SD (*n* = 3) from three independent experiments, analyzed by Student’s *t* test. *P < 0.05, **P < 0.01.

We initially determined the survival of patients with breast cancer according to TLR5 gene data using Kaplan–Meier plotter (http://kmplot.com/analysis/) and found significantly worse survival rates among patients with low TLR5 expression in tumors(Fig. 2a). Thereafter, CCK-8 assays showed that the ability to proliferate was higher for TLR5^−^ 4T1 than for TLR5^+^ 4T1 cells. The OD (450 nm) of TLR5^+^ 4T1 vs. TLR5^−^ 4T1 cells at 6, 12, 24, 36, and 48 h were 0.27 ± 0.01 vs. 0.26 ± 0.03, 0.28 ± 0.01 vs. 0.35 ± 0.02, 0.78 ± 0.04 vs. 1.01 ± 0.09, 1.19 ± 0.06 vs. 1.60 ± 0.05, and 1.78 ± 0.08 vs. 2.26 ± 0.07, respectively (Fig. 2b). Apoptosis in TLR5-downregulated 4T1 cells did not significantly change compared with the negative control (Fig. 2c, *P* *>* 0.05). We evaluated the ability of 4T1 cells to form tumors using colony formation assays to further determine the effects of TLR5 on tumor cell proliferation. Figure 2d shows that significantly more colonies were formed by TLR5^−^ 4T1 than by TLR5^+^ 4T1 cells, indicating higher tumorigenicity. The colony formation rates of TLR5^−^ 4T1 vs. TLR5^+^ 4T1 were 76 ± 8% vs. 59 ± 4% (*P* < 0.05), respectively. We verified differences in migration and invasiveness between TLR5^+^ 4T1 and TLR5^−^ 4T1 cells using wound healing and transwell assays. The migration area was larger in the TLR5^−^ 4T1, than the TLR5^+^ 4T1 group, and the migration area ratio of TLR5^+^ vs. TLR5^−^ 4T1 cells was 71.67 ± 3.05% vs. 55.33 ± 3.51% (*P* < 0.01; Fig. 2e). Transwell invasion assays showed that the number of migrated cells was remarkably lower in the TLR5^+^ 4T1 group than in the TLR5^−^ 4T1 group (35 ± 5 vs. 82 ± 15 cells/area; *P* < 0.01; Fig. 2f).

### TLR5 downregulation promoted 4T1 metastasis in vivo

**Fig. 3:**
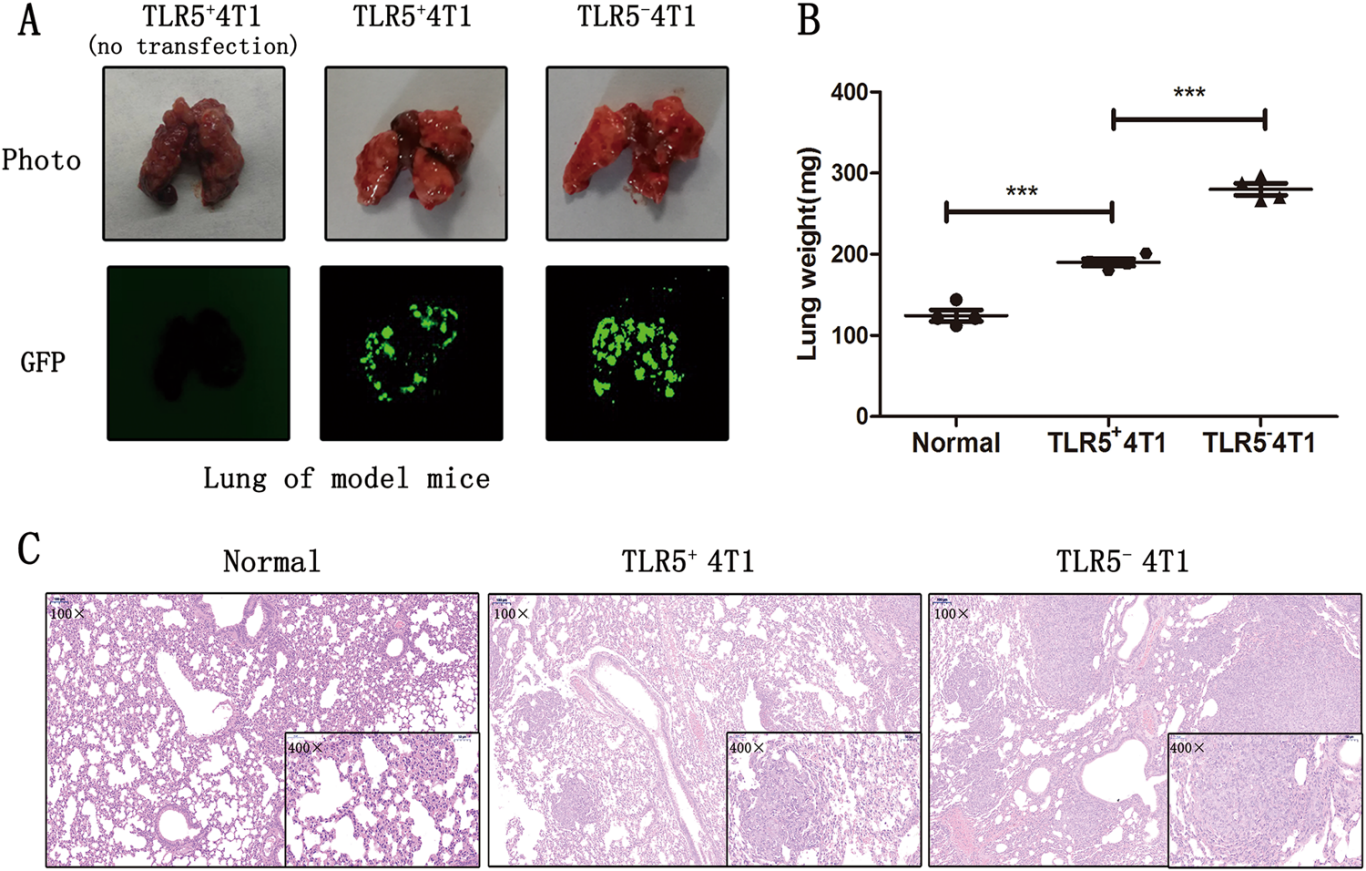
TLR5 downregulation promoted lung metastasis of 4T1 in vivo. Fluorescence images of stripped lungs of mice models: naive 4T1 (no Lentivirus transfection), TLR5^+^ 4T1 (with negative Lentivirus transfection) and TLR5^−^ 4T1 (with Lentivirus-shRNA TLR5 knockdown transfection) (**a**). Lungs from untreated mice, TLR5^+^ 4T1 and TLR5^−^ 4T1 model mice were weighed (**b**) and H&E staining was performed (**c**) (*n* = 3, **P < 0.01). Representative results of three independent experiments were reported. The data were presented as the means ± SD. The data were analyzed by Student’s *t* test. *P < 0.05, **P < 0.01.

At 21 days after injecting TLR5^+^ 4T1, TLR5^−^ 4T1 and non-transfected 4T1 cells via the tail vein, the lungs were stripped from the mice, weighed, and assessed by fluorescence imaging and histological analysis. Figure 3a shows much larger green fluorescence areas indicating metastatic sites in the TLR5^−^ 4T1 than in the TLR5^+^ 4T1 group, and green fluorescence was absent in the non-transfected 4T1 group. The lungs were the heaviest, averaging ~280 mg and reaching 300 mg, in the TLR5^−^ 4T1groups, whereas normal lungs weighed approximately 120 mg (Fig. 3b). Differences were quite apparent among the three groups (*P* *<* 0.05). Staining with H&E revealed typical tumor tissues changes within the lungs where fluorescence imaging positively. Tumor cells that metastasized to the lungs were closely arranged and had larger nuclei than those in normal lung tissues. The number and size of metastases were significantly smaller in the TLR5^+^ 4T1 than in the TLR5^−^ 4T1 group (Fig. 3c).

### Successful preparation of ^125^I-anti-TLR5 mAb and ^125^I-IgG

**Fig. 4:**
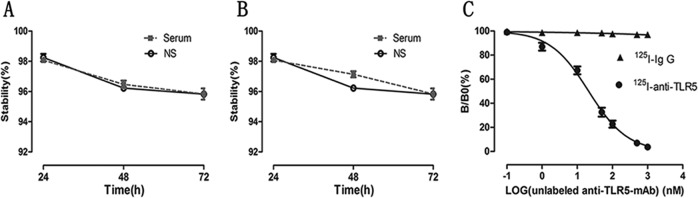
Evaluation of prepared radiolabeled tracers in vitro. The stability of ^125^I-antiTLR5 mAb (**a**) and ^125^I-IgG (**b**) in serum and normal saline changes over time. The concentration of the labeled tracer (^125^I-anti-TLR5 mAb or ^125^I-IgG) was kept constantly, and increasing concentrations of unlabeled anti-TLR5 mAb were used to compete with the ^125^I-anti-TLR5 mAb binding (**c**).

The radiochemical purity of ^125^I-antiTLR5 mAb and ^125^I-IgG was both >90%. Both tracers were stable at >95% up to 72 h and relatively stable in serum and normal saline with no detectable significant differences between them (Fig. 4a and B). Competitive binding analysis using >500-fold excess of unlabeled anti-TLR5 mAb almost completely blocked ^125^I-anti-TLR5 mAb (<5%) binding, with nonspecific binding for ^125^ I-IgG being ~2% (Fig. 4c).

### Dynamic phosphor-autoradiography and fluorescence imaging in vivo

**Fig. 5:**
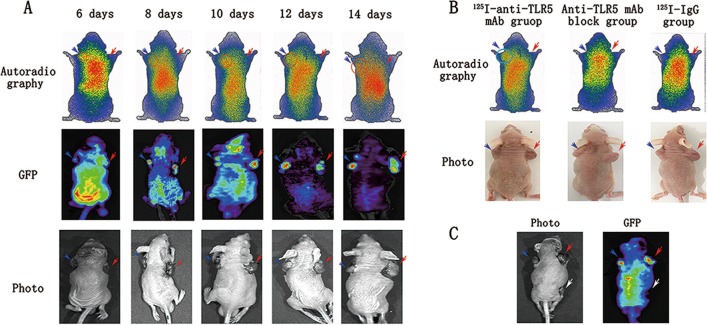
Dynamic phosphor-autoradiography and Fluorescence imaging. Representative images were performed at 48 h post-injection of ^125^I-antiTLR5 mAb/^125^I-IgG. The red arrow pointed to the TLR5^−^ tumor, and the blue was TLR5^+^ tumor and the white was TLR5^+^ without Lentivirus transfer (**a**–**c**). From day 6 to day 14, representative images of tumor-bearing mice in phosphor-autoradiography and fluorescence imaging (**a**). Representative phosphor-autoradiography images for the ^125^I-antiTLR5 mAb group, block group and ^125^I-IgG group (**b**). Representative images of fluorescence imaging (**c**). Representative results of three independent experiments were reported.

Dynamic phosphor-autoradiography and fluorescence imaging proceeded on day 6, 8, 10, 12, and 14 after the mice were subcutaneously injected with TLR5^+^ 4T1 and TLR5^−^ 4T1 cells and at 48 h after injecting them with ^125^I-anti-TLR5 mAb (Fig. 5a). Whole-body phosphor-autoradiography showed higher and lower radioactivity uptake by TLR5^+^ 4T1 and TLR5^−^ 4T1 tumors, respectively, at all time points. Dynamic phosphor-autoradiography on day 6 clearly showed TLR5^+^ 4T1 tumors, whereas TLR5^−^ 4T1 tumors were unclear. However, fluorescence imaging on day 6 clearly showed the location and boundary of TLR5^−^ 4T1 tumors, which might be due to the high specificity of ^125^I-anti-TLR5 mAb binding. The radioactivity (DLU)/mm^2^ was 6.67-fold higher in TLR5^+^ 4T1 than in TLR5^−^ 4T1 tumors (141,525 ± 8554 vs. 21,254 ± 2257 cpm). Autoradiography and fluorescence double imaging confirmed that TLR5 was a good reporter for noninvasive monitor and predictor of TNBC. Tumors were not obvious in specifically blocked and ^125^I-IgG groups at any checked time point, suggesting specific ^125^I-anti-TLR5 mAb accumulation in TLR5-positive tumors(Fig. 5b). Furthermore, to confirm that tumor fluorescence was GFP-tagged, transfected, virus-derived, and TLR5-specific, we inoculated mice with three types of tumors cells. The 4T1 tumors on the right and left flanks were transfected with lentivirus-TLR5 knockdown and negative-control Lentivirus, respectively, and the 4T1 tumor on the back was not transfected. Figure 5c clearly showed tumors in both flanks, whereas the non-transfected 4T1 tumor appeared black.

### Tumor imaging ex vivo

**Fig. 6:**
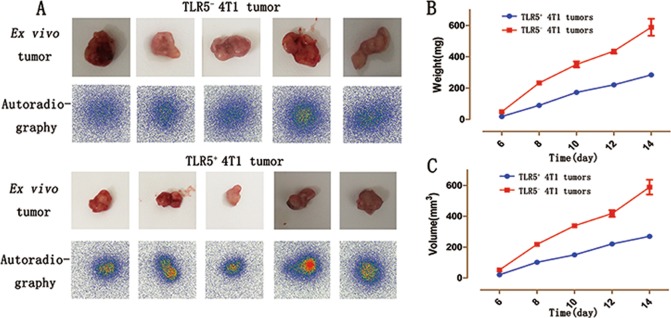
Isolated tumor in vitro phosphor-autoradiography imaging. TLR5^+^ 4T1 and TLR5^−^ 4T1 tumors were stripped and subjected to phosphor-autoradiography imaging (**a**), weighed (**b**) and volume measurement (**c**). Representative results of three independent experiments were reported. The data were presented as the means ± SD. The data were analyzed by Student’s *t* test. *P < 0.05, **P < 0.01.

Radioactivity accumulation was higher in isolated TLR5^+^ 4T1 than in TLR5^−^ 4T1 tumors visualized by imaging ex vivo (Fig. 6a). Furthermore, the weight and volume were much larger for TLR5^−^ 4T1 than for TLR5^+^ 4T1 tumors (Fig. 6b, c; *P* < 0.05).

### Biodistribution studies

To validate the imaging results and further quantify ^125^I-antiTLR5 mAb uptake at 24, 48, and 72 h, biodistribution studies in vitro proceeded 14 days after TLR5^+^ 4T1 and TLR5^−^ 4T1 tumor cell inoculation. Table 1 showed that the T/NT (target to non-target) ratio was the highest in TLR5^+^ 4T1 tumors at 48 h. Targeting by the ^125^I-antiTLR5 mAb was more efficient in TLR5^+^ 4T1 than in TLR5^−^ 4T1 tumors. The uptake of ^125^I-antiTLR5 mAb in TLR5^+^ 4T1 tumors at 24, 48, and 72 h post-injection were 7.73 ± 0.75, 4.92 ± 0.36, and 2.58 ± 0.18 (%ID/g), respectively, with T/NT ratios of 6.84 ± 1.04, 8.41 ± 0.91, and 7.15 ± 1.8, respectively, whereas ^125^I-antiTLR5 mAb uptake in TLR5^−^ 4T1 tumors was 1.39 ± 0.1, 0.7 ± 0.05, and 0.42 ± 0.03 (%ID/g), respectively, with T/NT ratios of 1.16 ± 0.06, 1.21 ± 0.15, and 1.15 ± 0.13, respectively. The T/NT ratio of the ^125^I-antiTLR5 mAb was significantly lower for TLR5^−^ 4T1 than for TLR5^+^ 4T1 tumors. In addition, that of ^125^I-IgG was only 1.48 ± 0.23 at 48 h, suggesting nonspecific ^125^I-IgG tumor binding. In the block group, the ^125^I-anti-TLR5 mAb uptake of TLR5^+^ 4T1 tumor was only 1.67 ± 0.14 %ID/g at 48 h, suggesting that ^125^I-antiTLR5 binding with tumor was blocked by unlabeled anti-TLR5 mAb pre-injection in vivo. At 48 h, the T/L (tumor to liver), T/K (tumor to kidney), and T/B (tumor to blood) ratios were significantly higher for the TLR5^+^ 4T1 tumor than for the TLR5^−^ 4T1 tumor (1.70 ± 0.18 vs. 0.24 ± 0.02, 1.68 ± 0.28 vs. 0.24 ± 0.04, and 1.73 ± 0.12 vs. 0.25 ± 0.02, respectively; *P* < 0.01 for all). Background radioactivity in all other organs and tissues was minimal, which was in agreement with the imaging data.

**Table 1:** Biodistribution of ^125^I-anti-TLR5 mAb in TLR5^+^ and TLR5^−^ 4T1 tumor models group.

	Time (h)		
	24	48	72
Blood	4.47 ± 0.55	2.87 ± 0.41	1.42 ± 0.12
Thyroid	2.28 ± 0.13	1.15 ± 0.31	1.46 ± 0.40
Heart	2.02 ± 0.32	1.63 ± 0.09	1.15 ± 0.12
Lung	5.00 ± 0.87	1.31 ± 0.31	1.23 ± 0.14
Liver	7.56 ± 0.71	2.91 ± 0.23	2.05 ± 0.12
Spleen	4.05 ± 0.99	0.86 ± 0.07	0.66 ± 0.11
Kidney	8.36 ± 1.02	3.02 ± 0.77	1.38 ± 0.20
Intestines	1.41 ± 0.20	0.54 ± 0.22	0.59 ± 0.37
Bone	0.85 ± 0.26	0.61 ± 0.15	0.29 ± 0.05
Muscle	1.20 ± 0.14	0.59 ± 0.11	0.37 ± 0.06
Tumor(TLR5^+^)	7.73 ± 0.75**	4.92 ± 0.36**	2.58 ± 0.18**
Tumor(TLR5^−^)	1.39 ± 0.10	0.70 ± 0.05	0.42 ± 0.03

^*125*^*I* radiolabeled sodium iodide, *h* hours

**P < 0.01 compared to the TLR5^+^ 4T1 tumor and the TLR5^−^ 4T1 tumor in the same mouse. Data are presented as a mean percent injected dose per gram (%ID/g) ± standard deviation (Mean ± SD) of five animals

### H&E and immunohistochemical staining

**Fig. 7:**
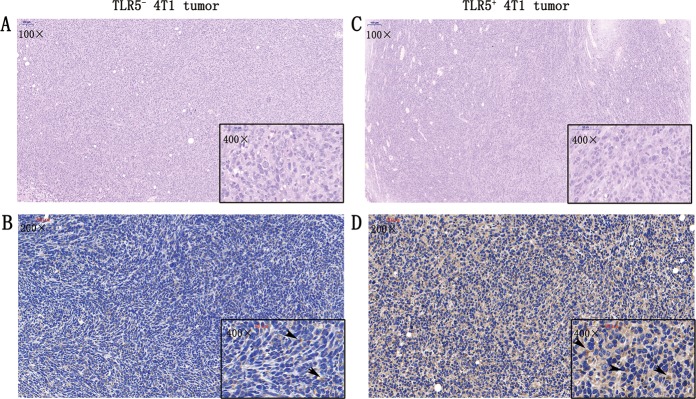
H&E staining and immunohistochemistry staining. For TLR5^−^ 4T1 tumors, representative microscopy images H&E (**a**) and immunohistochemistry staining for TLR5 (**b**), black arrow refers to the positive area (brown) (**a**). For TLR5^+^ tumors, representative microscopy images the H&E staining (**c**) and immunohistochemistry staining for TLR5 (**d**), black arrow refers to the positive area (brown). *n* = 5, **p < 0.01. Representative results of five independent experiments were reported. The data were presented as the means ± SD. The data were analyzed by Student’s *t* test. *P < 0.05, **P < 0.01.

Figure 7a, c show representative microscopy images of TLR5^−^ 4T1 and TLR5^+^ 4T1 tumor sections (5-μm-thick) stained with H&E (100× and 400× magnification) and immunohistochemically stained for TLR5 (200× and 400× magnification) (Fig. 7b, d). Staining with H&E showed typical changes in tumor tissues. The surface and cytoplasm of tumor cells were immunohistochemically stained brown indicating TLR5 positivity. The ratio of positively stained tumor cells was much higher for TLR5^+^ 4T1 than for TLR5^−^ 4T1 tumors (*n* = 5; 68.75 ± 5.25% vs. 20.65 ± 5.65%, *P* < 0.01).

## Discussion

This study showed that TLR5 downregulation prompted TNBC proliferation, metastasis, and invasion, in vitro and in vivo. More importantly, we found that TLR5 on TNBC could potentially serve as a reporter for lower metastasis, lower invasion, and better prognosis. Our results revealed a novel target molecule for earlier TNBC detection and prognosis prediction, and this could provide the means for new strategies to monitor and predict TLR5-positive tumors.

Dynamic interactions between tumors and their microenvironments are essential for tumor growth, angiogenesis, and metastasis^29^. High levels of TLR5 were expressed during intestinal epithelial cell-mediated anti-tumor activity in a mouse xenograft model of human colon cancer^30^. Our findings are consistent with these results. To understand whether the downregulation of TLR5 could promote breast cancer metastasis and invasion, we detected EMT markers and its signal pathway, TRAF6 and SOX2. The EMT represented a fatal transfer of cancer progression^31^. Twist1, a transcription factor, played a key role in cancer development and progression^32^ and up-regulated Twist1 induced EMT and E-cadherin repression, indicating that Twist1 promoted metastasis by inducing EMT^33^. Using western bolt and qPCR, we found E-cadherin and TLR5 expression decreased in TLR5^−^ 4T1 cells, whereas vimentin, fibronectin, N-cadherin and Twist1 expression increased. The metastatic and invasive abilities of TLR5^−^ 4T1 cells were enhanced. Notably, The expression of TRAF6 and SOX2 increased, and it was reported that elevated TRAF6 expression prompted SOX2 expression^34^, and upregulation of SOX2 prompted tumor metastasis through EMT, in breast cancer and other kind of tumors^35,36^. These results might help to further understand why downregulated TLR5 expression promoted 4T1 cell proliferation, metastasis, and invasion. Our data in vitro showed that TLR5 expressed in breast cancer could serve as a reporter of cancer invasiveness.

Bioprospecting for target molecules represents a promising solution for cancer prognosis^37,38^. TNBC is associated with aggressive tumor behavior and a worse prognosis^39^. However, the early discovery of TNBC is particularly important, since its onset, early metastasis, and aggressive invasiveness is coupled with a lack of targeted therapy^40–42^. Therefore, identifying novel targets that closely correlate with TNBC progression is of considerable importance for its monitor and prognosis.

We previously correlated TLR5 expression with allo-transplant rejection^28^. The expression of TLR5 in NK cells from patients with breast cancer has recently been discovered^43^, and TLR5 expression could restrain tumor growth and metastasis in vitro and in vivo^44^. Based on these findings, we postulated that TLR5 in TNBC cells might play an important role in the progression of TNBC and serve as a novel target suitable for early TNBC monitor and prediction.

Targeted radioimmunoimaging with monoclonal antibodies against many kinds of cancers has been successful^24^. Radiolabeled ^125^I-antiTLR5 mAb might provide a means of visualizing TLR5 expression in vivo. Our data suggested that ^125^I-antiTLR5 mAb, which is clinically relevant, can be used in TNBC tumor models.

Our results in vitro indicated that TLR5 in TNBC was closely associated with cell invasion, but whether the same behavior retained in vivo remains unknown. We therefore established a xeno-subcutaneous tumor model using nude mice, then monitored with an ^125^I-labeled anti-TLR5 mAb tracer, which was with high affinity for TLR5 in 4T1 cells in vitro. The distribution in vitro and results of whole-body phosphor-autoradiography in vivo revealed much higher radiotracer retention in TLR5^+^ 4T1 than in TLR5^−^ 4T1 tumors at all time points. The ^125^I-antiTLR5 mAb could not target TLR5^+^ 4T1 tumor in group of pre-injecting unlabeled anti-TLR5 mAb, and tumors were not visualized by radiography, thus, confirming the specificity of ^125^I-antiTLR5 mAb imaging.

Fluorescent reporter gene imaging in vivo has been applied to detect the growth and migration of labeled cells in many kinds of diseases^45^. Here, we monitored tumor locations using a TLR5 knockdown 4T1 cell line labeled with green fluorescent protein. The fluorescence images of virus-transfected 4T1 tumors were much clearer than those of non-transfected 4T1 tumors. Moreover, this method had the advantage of clearly displaying tumor margins. More importantly, we found that locations of fluorescence emission contained more tracer radioactivity in TLR5^+^ 4T1 tumor, which further confirmed that tumor imaging was TLR5 expression-specific. Additionally, early images showed the TLR5^−^ 4T1 tumors were larger than TLR5^+^ 4T1 tumors, which suggested that TLR5 expression might inhibit tumor growth in vivo.

To confirm that TLR5 affects tumor metastasis, we established lung metastasis mouse models by injecting TLR5^+^ 4T1 and TLR5^−^ 4T1 cells via tail veins, and evaluated the findings using fluorescence imaging. The results showed much clearer lung tumors in TLR5^−^ 4T1 group than in TLR5^+^ 4T1 group, suggesting that TLR5 negatively affected TNBC metastasis.

These data suggested that TLR5 expressed in breast cancer could be considered as a biomarker in vivo for the noninvasive molecular imaging of TNBC. It could also be used to monitor tumor development and metastasis, predict its prognosis, evaluate therapy responses, and as a target for therapy. Low tracer uptake in the lungs and heart, and high uptake in tumors increased image contrast. These cell-specific and favorable non-target clearance features of ^125^I-antiTLR5 mAb rendered it a promising radiotracer for TNBC imaging.

This study had some limitations. The molecular weight of labeled antibodies was too high for clinical applications. This might be overcome by reducing extended circulation by selecting antibody fragments, adapters, or other small molecules that specifically bind to TLR5. A radioisotope-labeled TLR5-targeting probe suitable for clinical application, such as iodine 131 or the positron nuclide Fluoro-18, should be further investigated. Hence, additional analyses of TLR5 on breast cancer in vitro and in vivo are required.

In conclusion, the downregulation of TLR5 in TNBC increased tumor invasiveness and EMT expression and promoted TNBC metastasis. Therefore, TLR5 might be useful for monitoring and evaluating TNBC prognosis and serve as a novel target for the early detection of TNBC and other TLR5-positive tumors. The radioisotope-labeled probe, ^125^I-antiTLR5 mAb, could potentially serve as an ideal noninvasive monitoring agent for TLR5-positive tumors.

## Supplementary information

Supplemental material 1

Supplemental material 2

Supplemental material 3

supplemental materials' legends

Reporting Checklist

Declaration of contributions to article
