# Supplementary figures and images for: TLR5: A prognostic and monitoring indicator for triple-negative breast cancer
Source: Cell Death Dis. 2019 Dec 18;10(12):954. doi: 10.1038/s41419-019-2187-8 (PMC6920449; doi:10.1038/s41419-019-2187-8)

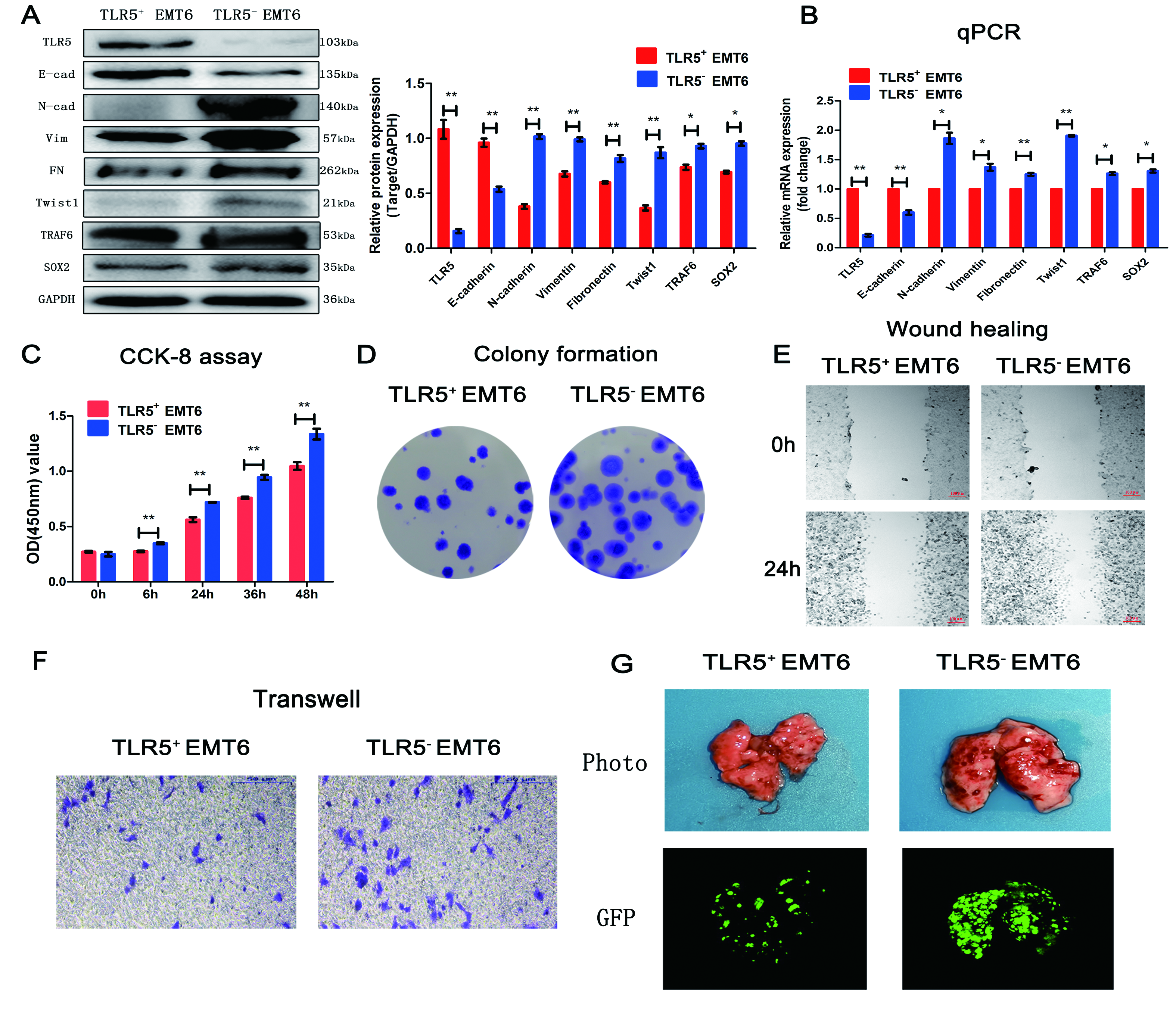

Supplement: Supplementary file 1 — Supplemental material 1 [file 41419_2019_2187_MOESM1_ESM.tif]

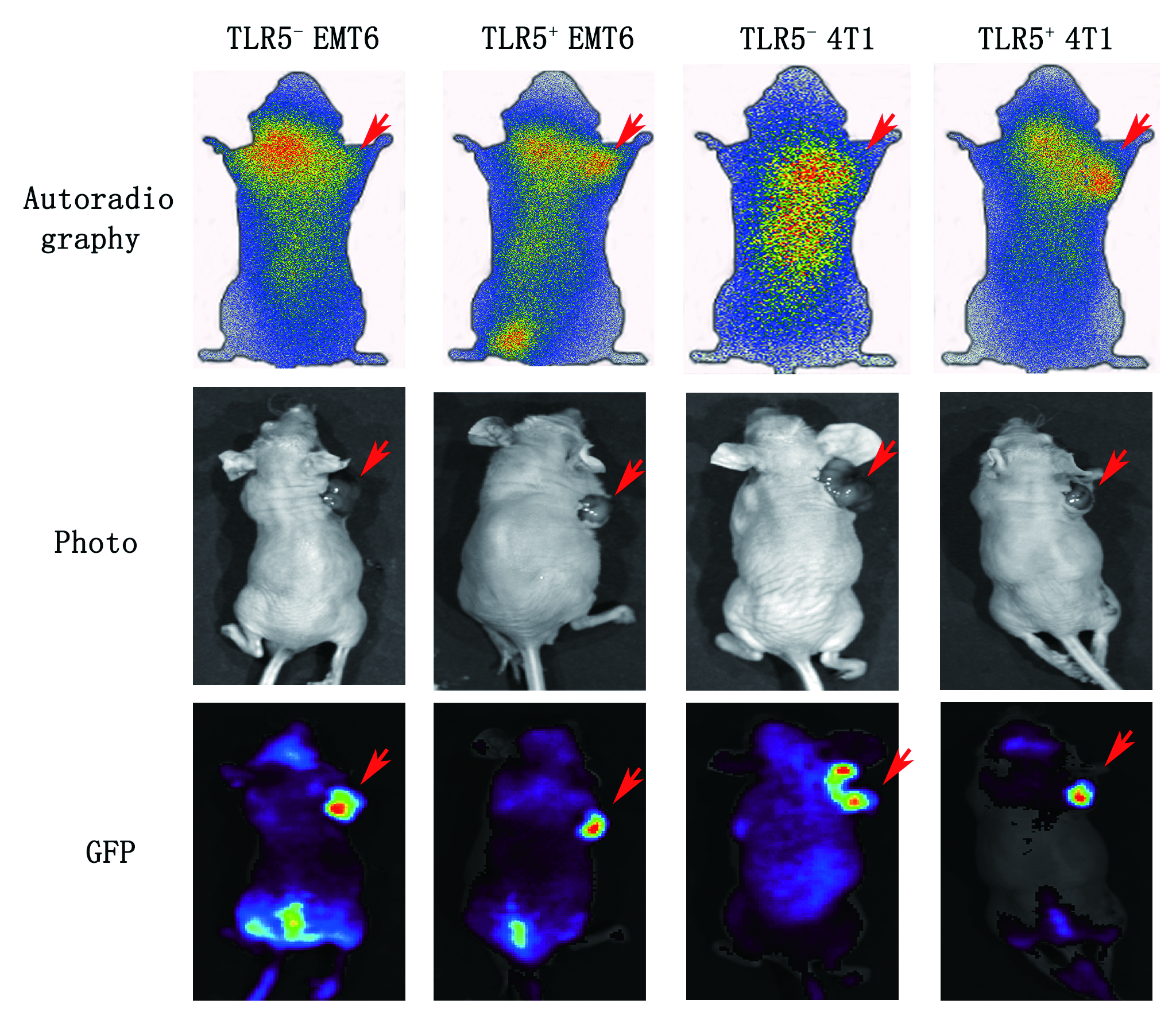

Supplement: Supplementary file 2 — Supplemental material 2 [file 41419_2019_2187_MOESM2_ESM.tif]

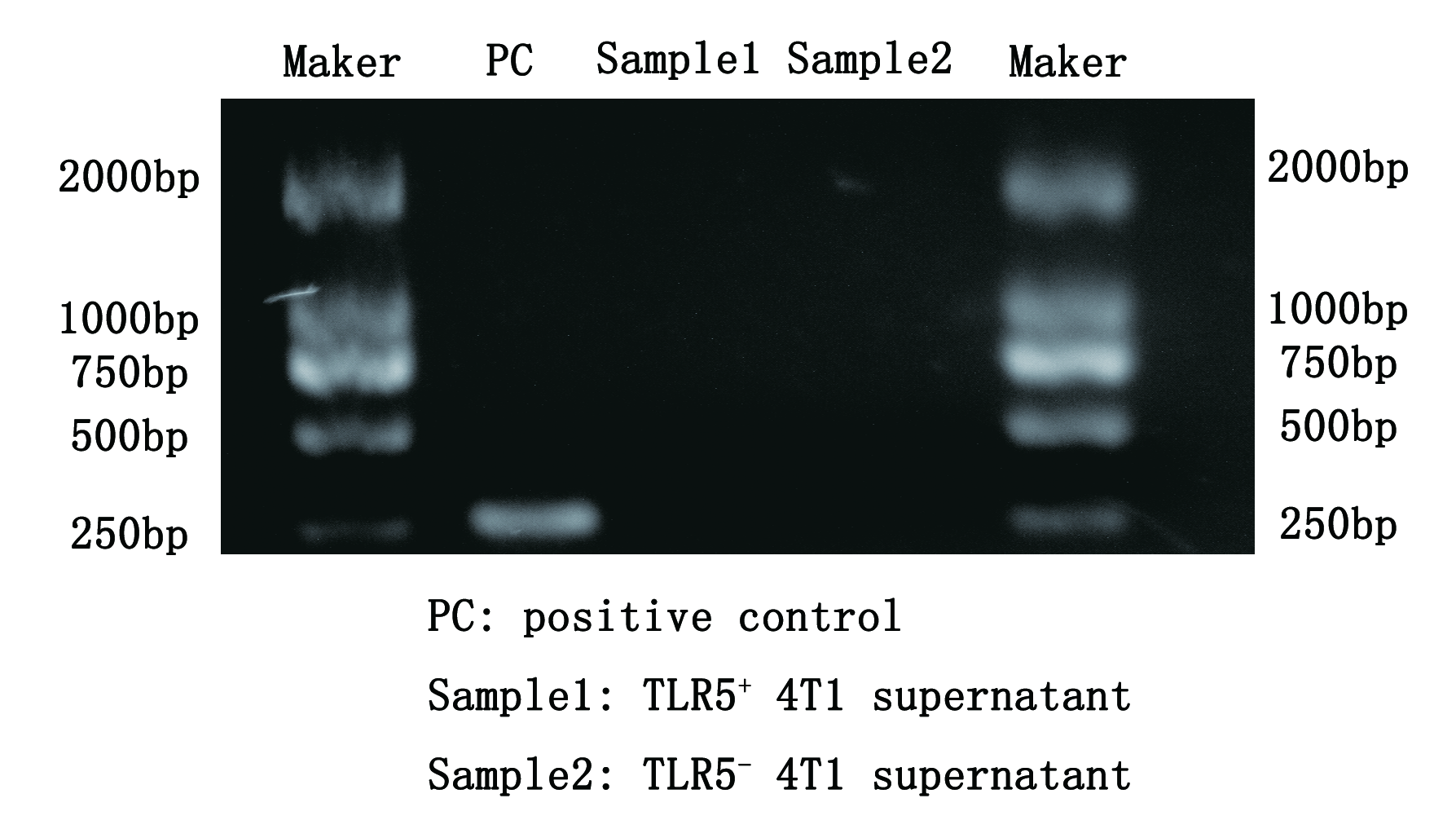

Supplement: Supplementary file 3 — Supplemental material 3 [file 41419_2019_2187_MOESM3_ESM.tif]

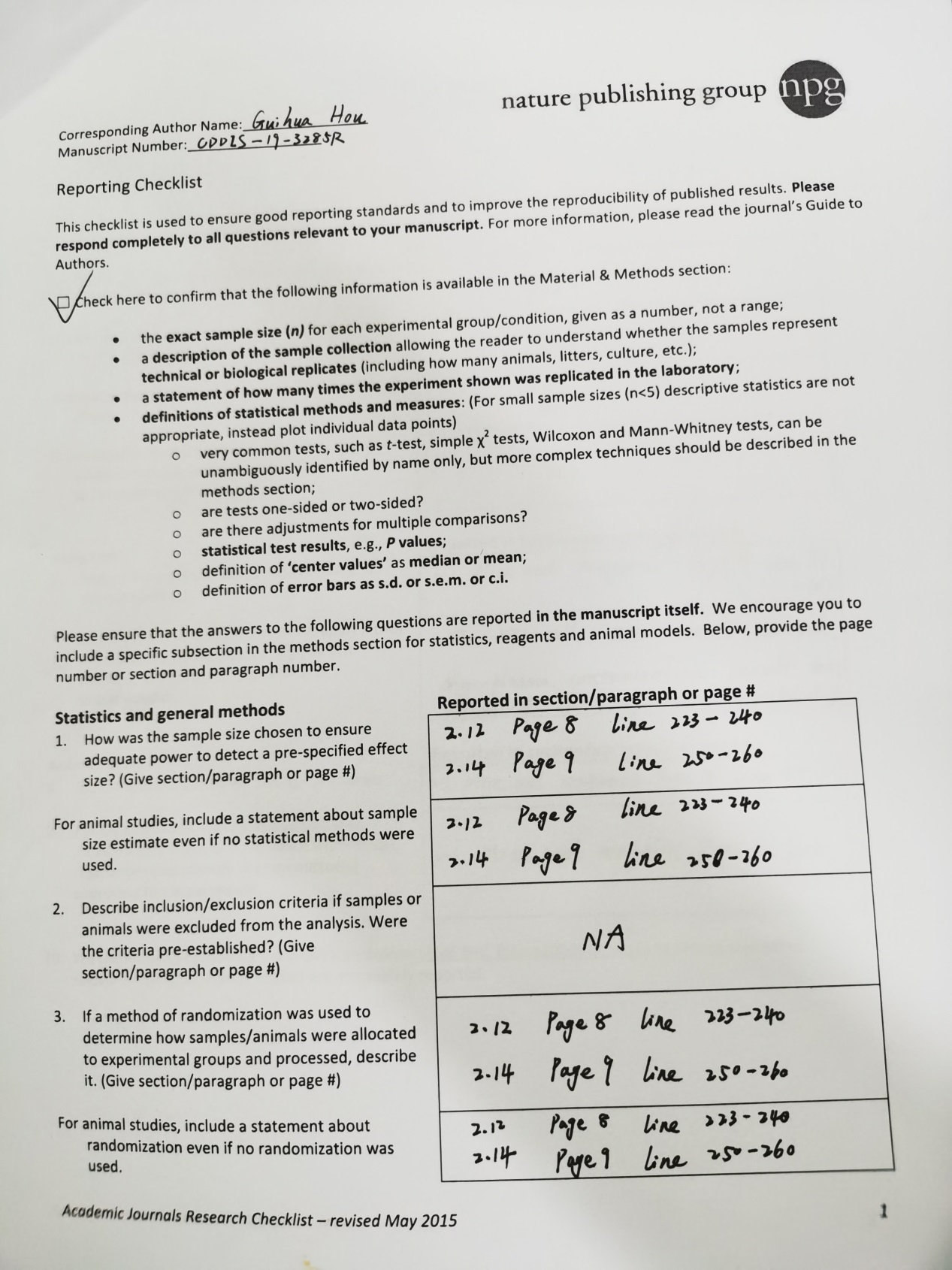


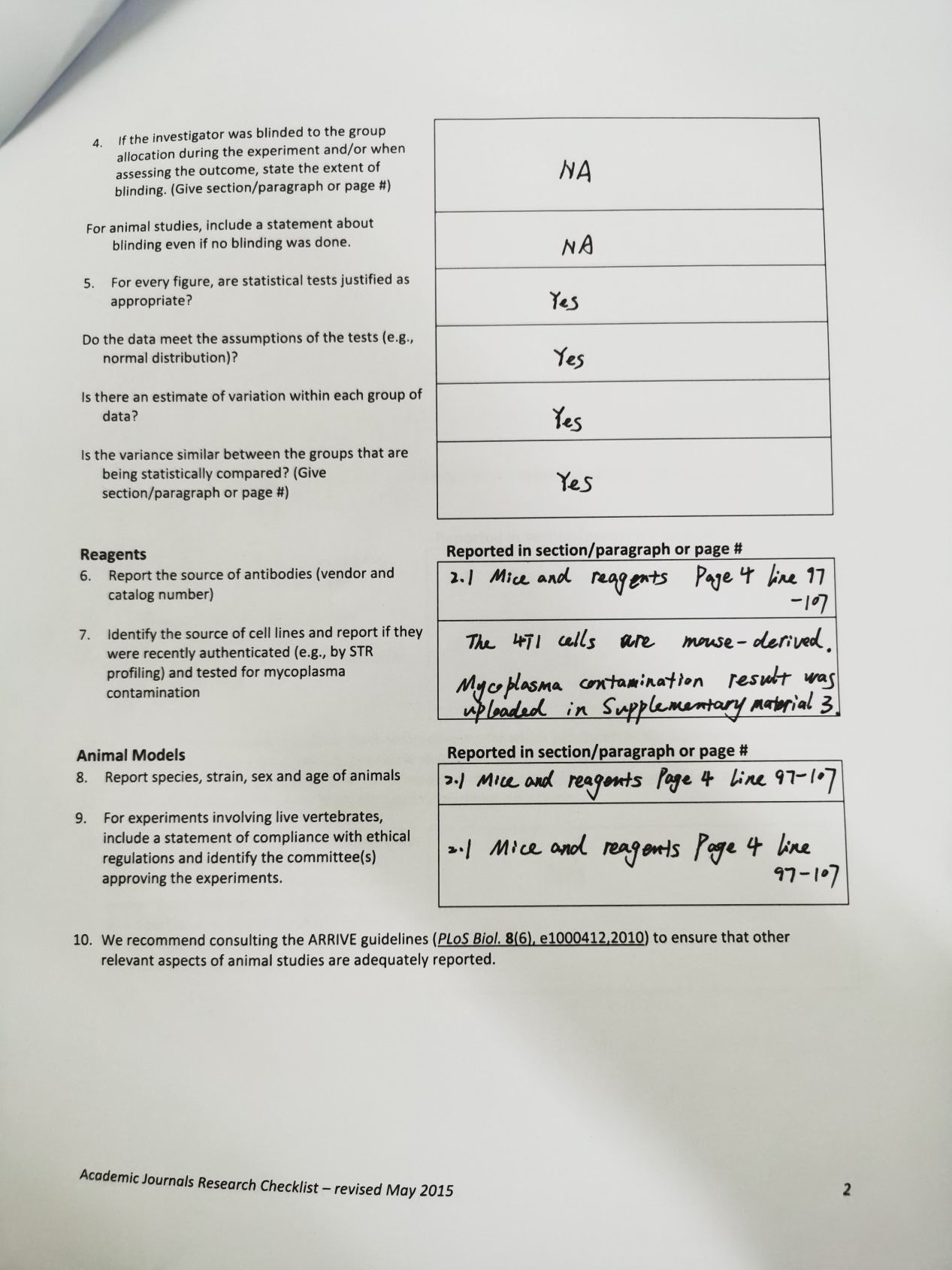

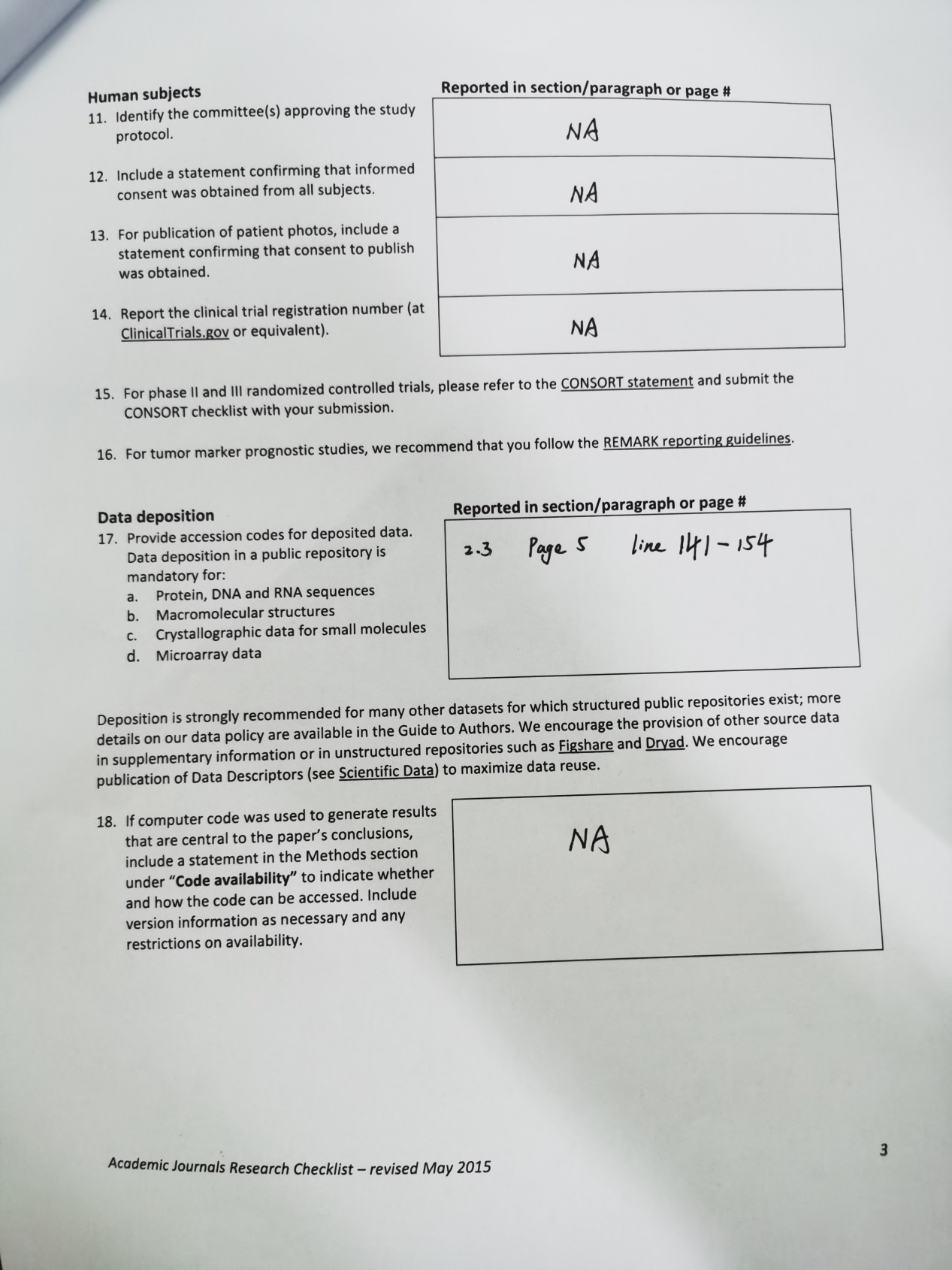

Supplement: Supplementary file 5 — Reporting Checklist [file 41419_2019_2187_MOESM5_ESM.docx]

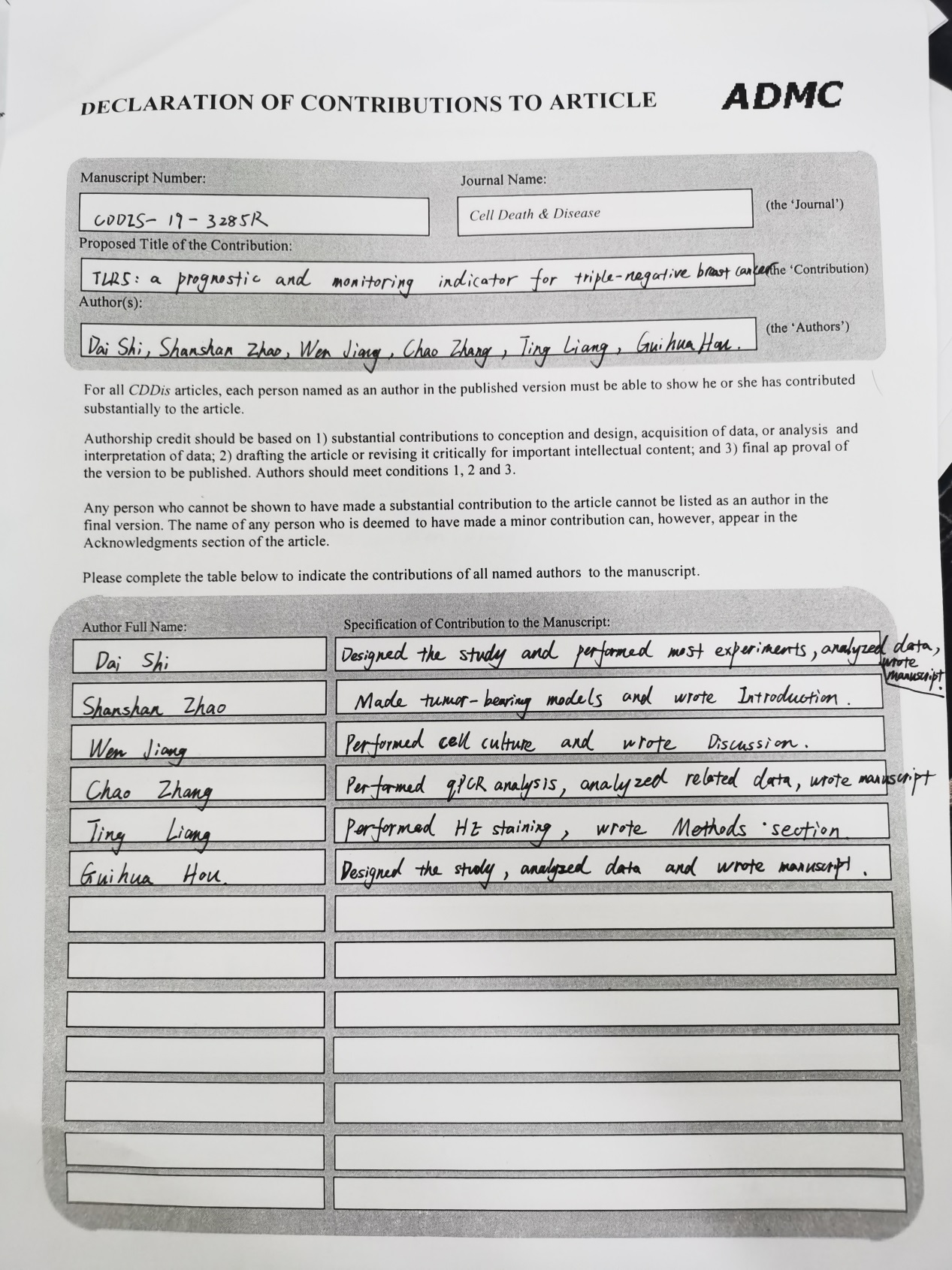

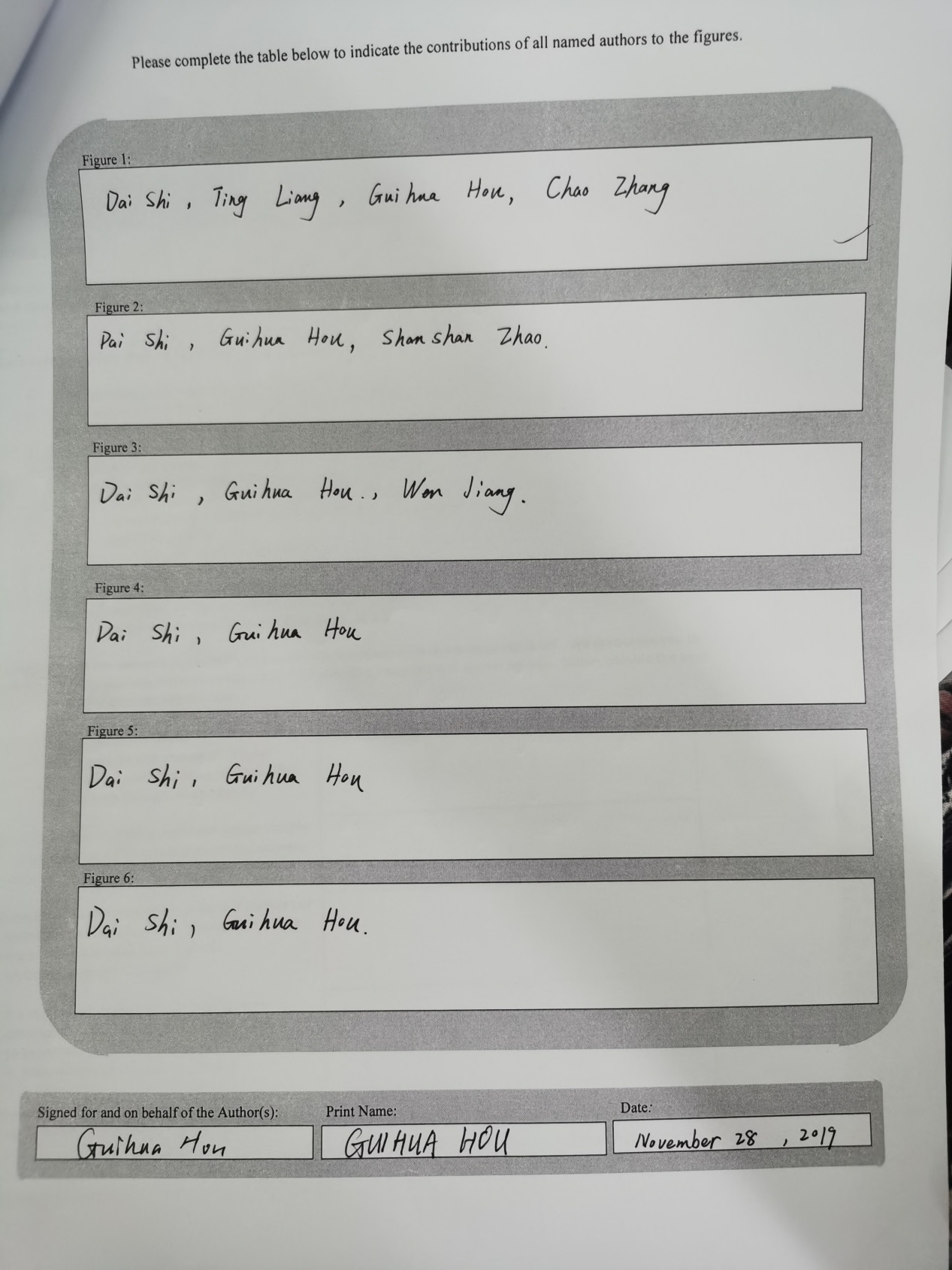

Supplement: Supplementary file 6 — Declaration of contributions to article [file 41419_2019_2187_MOESM6_ESM.docx]
