# Supplementary material for: TLR5: A prognostic and monitoring indicator for triple-negative breast cancer
Source: Cell Death Dis. 2019 Dec 18;10(12):954. doi: 10.1038/s41419-019-2187-8 (PMC6920449; doi:10.1038/s41419-019-2187-8)
Supplement: Supplementary file 4 — supplemental materials' legends [file 41419_2019_2187_MOESM4_ESM.docx]

Supplement materials 1. TLR5 down-regulation promoted EMT and enhanced EMT6 tumor cell proliferation, migration and invasiveness *in vitro* and in vivo. TLR5, E-cadherin, N-cadherin, vimentin, fibronectin, TRAF6, SOX2 and Twist1 protein expression of TLR5^+^ EMT6 and TLR5^-^ EMT6 cells was detected by western Blot (n=3, **P*<0.05) (A). TLR5, E-cadherin, N-cadherin, vimentin, fibronectin, TRAF6, SOX2 and Twist1 mRNA expression of TLR5^+^ EMT6 and TLR5^-^ EMT6 cells was detected by qPCR (n=3, **P*<0.05) (B). CCK-8 assay showed that TLR5^-^ EMT6 cells have higher proliferation ability than TLR5^+^ EMT6 cells (C). The colony formation ability of TLR5^-^ EMT6 cells was significantly increased than TLR5^+^ EMT6 cells (D).The migration area of TLR5^-^ EMT6 group was larger than TLR5^+^ EMT6 group (E). The number of migrated cells was remarkably reduced in TLR5^+^ EMT6 group than TLR5^-^ EMT6 group (F). TLR5 down-regulation promoted lung metastasis of EMT6 tumors *in vivo* (G). The data were presented as the means ± SD (n=3) from three independent experiments, analyzed by Student’s t test. **P*<0.05, ***P*<0.01.

## Supplement materials 2. Dynamic phosphor-autoradiography and Fluorescence imaging. Representative images were performed at 48h post-injection of ^125^I-antiTLR5 mAb. The red arrow pointed to tumors’ location. At the day 10 after inoculating different kinds of tumor cells, representative images of tumor-bearing mice in phosphor-autoradiography and fluorescence imaging.

Supplement materials 3. Mycoplasma contamination detection of TLR5^+^ and TLR5^-^ 4T1.
